# Meeting the challenge of teaching bioethics: a successful residency curricula utilizing Team-Based Learning

**DOI:** 10.1080/07853890.2021.2013523

**Published:** 2022-02-04

**Authors:** Sandra P. Spencer, Stephanie Lauden, Sheria Wilson, Andrew Philip, Rena Kasick, John D. Mahan, Ashley K. Fernandes

**Affiliations:** a Division of Emergency Medicine, Nationwide Children’s Hospital, Boston, MA, USA; b Department of Pediatrics, The Ohio State University College of Medicine, Columbus, OH, USA; c Division of Hospital Pediatrics, Nationwide Children’s Hospital, Columbus, OH, USA; d Division of Neonatology, Nationwide Children’s Hospital, Boston, MA, USA; e Department of Psychiatry, The Ohio State College of Medicine, Columbus, OH, USA; f Division of Nephrology, Nationwide Children’s Hospital, Boston, MA, USA; g Division of Primary Care Pediatrics, Nationwide Children’s Hospital, Boston, MA, USA; h Center for Bioethics, The Ohio State University College of Medicine, Columbus, OH, USA

**Keywords:** Bioethics, bioethics education, graduate medical education, medical ethics education, paediatric bioethics, professionalism education, residency curriculum, residency education, significant learning, Team-Based Learning (TBL)

## Abstract

**Background:**

Despite recognition by both the Accreditation Council of Graduate Medical Education (ACGME) and the American Board of Paediatrics (ABP) of the importance of bioethics education, curricular crowding, lack of perceived significance, and insufficient administrative support remain significant barriers to trainees gaining competency in bioethics. Few bioethics curricula at the graduate medical education level are evidence-based or comprehensive. We sought to develop and assess the effectiveness of a Team Based Learning (TBL) curriculum in improving residents’ bioethics knowledge and their ability to evaluate ethical dilemmas.

**Methods:**

We integrated L. Dee Fink’s curricular design principles of “Significant Learning,” Jonsen *et. al*’s “Four-Box Method” of ethical analysis, and ABP bioethics content specifications to create 10 TBL bioethics sessions. Paediatric residents at a major academic centre then completed a 3-year longitudinal, integrated TBL-based bioethics curriculum. Primary outcomes included individual and group readiness assessment tests (iRAT/gRAT), pre-work completion, and satisfaction with sessions.

**Results:**

The TBL-based bioethics curriculum contains 10 adaptable modules. Paediatric residents (*n* = 348 total resident encounters) were highly engaged and satisfied with the curriculum. gRAT scores (mean 89%) demonstrated significant improvement compared to iRAT scores (72%) across all TBLs and all post-graduate years (*p* < .001). Higher gRAT scores correlated with higher level of training. Although pre-work completion was low (28%), satisfaction was high (4.42/5 on Likert scale).

**Conclusions:**

Our TBL-based bioethics curriculum was effective in improving knowledge, practical and flexible in its implementation, and well-received. We attribute its success to its grounding in ethical theory, relevance to ABP specifications, and a multi-modal, engaging format. This curriculum is easily modified to different specialties, virtual formats, or other specific institutional needs.

**Key messages**
- Despite formidable challenges to teaching bioethics in residency education, evidence-based methods such as Team-Based Learning (TBL) can be employed to increase knowledge and satisfaction.
- This study reports the first successful TBL bioethics curriculum, planned and executed longitudinally over 3 years, with paediatric residents at a large academic children’s hospital in the US.
- TBL can be utilised to teach bioethics at the graduate medical education level and is adaptable to different situational factors, disciplines, and levels of clinical experience.

## Background

Physicians face complex ethical and professional challenges as they weigh the autonomy and best interests of the patient, all within the context of the dynamic patient-family-physician triad. Complicating matters, laws guiding application of basic bioethical principles vary between states. Families and healthcare professionals are adapting to ever-changing advancements in technology and life-prolonging interventions. Within the field of paediatrics, both the Accreditation Council for Graduate Medical Education (ACGME) and the American Board of Paediatrics (ABP) recognise the importance of providing instruction and evaluation of competency in ethics and professionalism to trainees. Consequently, the ABP explicitly identified bioethical principles required for competency in paediatrics, and the ACGME requires paediatric residency programs to provide a structured bioethics and professionalism curriculum [1,2].

Despite mandates and the recognition that the application of bioethical principles are essential skills for any physician, guidelines for effectively teaching bioethics and professionalism are yet to be firmly established, and to our knowledge no study to date has compared the effective paediatric bioethics curricula with each other to determine best practices [3]. A recent systematic review of bioethics education found few validated bioethics curricula, and existing options varied in both content and effectiveness [3–8]. The American Academy of Paediatrics (AAP) Section on Bioethics created a 20-module, online, case-based bioethics curriculum with teaching guides to help address this need [9]. However, one study showed poor national implementation among residency programs (15%) [8]. Another review of paediatric bioethics education concluded that “existing training regimens are insufficient to meet the real-life ethical challenges experienced in actual practice, particularly with respect to palliative care and the commission of clinical errors.” (p. e66) [10].

While many paediatric residency programs offer some formal ethics education, broad implementation of ethics-related curricula represents another challenge [11]. Within the constraints of graduate medical education (GME), bioethics education competes with required clinical or educational duties. “Crowding in the curriculum” is an acknowledged barrier to curricular innovations in GME [8]. Lack of faculty expertise, challenges in engaging trainees, and minimal administrative support represent additional obstacles [8,12,13].

Lastly, best practices in evaluating the efficacy and applicability of bioethics curricula remains ill-defined. One study found 53% of residency programs had no formal assessment of clinical ethics knowledge and/or skills in their programs [8]. Although programs utilised observation, examinations, and simulated patients to assess resident bioethics comprehension, knowledge of ethical principles may not necessarily translate into the ability to *apply* these principles in complicated patient care situations [8].

**Figure 1.**
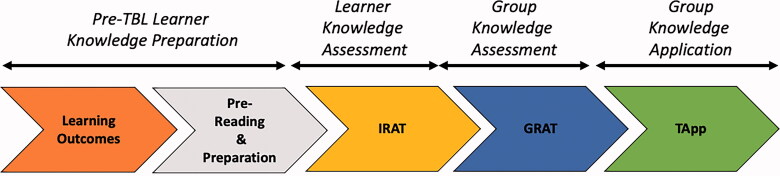
The team based learning process [21]. iRAT: Individual Readiness Assurance Test: a brief multiple-choice quiz to assess learners on knowledge necessary to achieve learning outcomes; gRAT: Group Readiness Assurance Test: the iRAT test is taken collaboratively within a small group, with immediate feedback given as to the correct answer once a group commits by consensus; a discussion of learning points follows, facilitated by faculty; TApp: Team Application Exercise: small groups of learners work through an ethical case/common problem together and arrive at an answer by consensus; each group reveals its answer simultaneously; faculty facilitate inter-group discussion surrounding ethical points of conflict.

With these complexities and barriers in mind, our Bioethics Education Task Force was created (2015) with the support of the Chair of Paediatrics and the Residency Program Director. First, we conducted a literature review of bioethics medical education best practices. In our analysis of evidence-based teaching methodologies, Team-Based Learning (TBL) was pedagogically appealing for several reasons. TBL is widely recognised for its ability to effectively facilitate knowledge acquisition in a variety of settings, and the promotion of significant learning through both active discussion and immediate application of complex concepts [14]. North American medical schools commonly use TBL, where it is effective in both clinical and pre-clinical settings [15–20]. Using TBL yields higher learner satisfaction and engagement compared to more traditional teaching modalities [21]. GME-focussed TBL approaches have been effective in internal medicine, primary care, and pathology training programs [22–26]. Briefly, TBL methodology is composed of (1) learning outcomes; (2) pre-TBL preparation; (3) an Individualised Readiness Assurance Test (iRAT); (4) a Group Readiness Assurance Test (gRAT); and (5) a Team Application Exercise (TApp), where learners *practice* the knowledge gained from the preceding steps (Figure 1).

Despite its demonstrated success, we found only three studies utilising TBL to teach bioethics. One evaluated a single TBL, and another evaluated only medical students. The third is our initial report, which provides a full description of the theoretical basis and educational process we utilised for creating this TBL curricula and presents the qualitative analysis which showed high learner satisfaction [27–29].

Here, we present our TBL-based bioethics curriculum and highlight its quantitative results. We hypothesised that a comprehensive bioethics curriculum using TBL, designed for paediatric residents, would improve knowledge acquisition, and allow for application of bioethical concepts. Our study sought to demonstrate knowledge gains in paediatric trainees with clinical experience, in the context of a three-year integrated bioethics curriculum.

## Methods

### Phase 1: Curricular planning

This TBL-based bioethics curriculum was designed for paediatric residents at a large, free standing, urban, tertiary care paediatric hospital with approximately 50 paediatric and internal medicine/paediatric residents per class. TBL module development followed the principles outlined in “Team-Based Learning: A Practical Guide,” [21] with two notable exceptions related to team dynamics: the use of organising learners into permanent teams, and peer review after the TBL. While we acknowledge the advantages of both in trust-building, collaboration, and accountability, the unique nature of medical residency’s rotating clinical demands and heterogenous curricular requirements (e.g. no formal “grades”) made permanent teams and peer assessments less than ideal. Instead, we presumed that our resident learners entered sessions having already extensively worked in (clinical) teams requiring trust, accountability, and peer feedback, and this would be a sufficient basis for success given our logistical realities. A comprehensive description of the process and theoretical basis employed has been published elsewhere [29]. Residents were exposed to the entire curriculum over a three-year period, with bioethics TBLs presented approximately three times a year during protected resident medical educational sessions. This study was deemed exempt by the Nationwide Children’s Hospital institutional review board (STUDY00000768, exempted since the data collected and analysed was part of the required curriculum, which would have been collected regardless for tracking and quality improvement).

**Figure 2.**
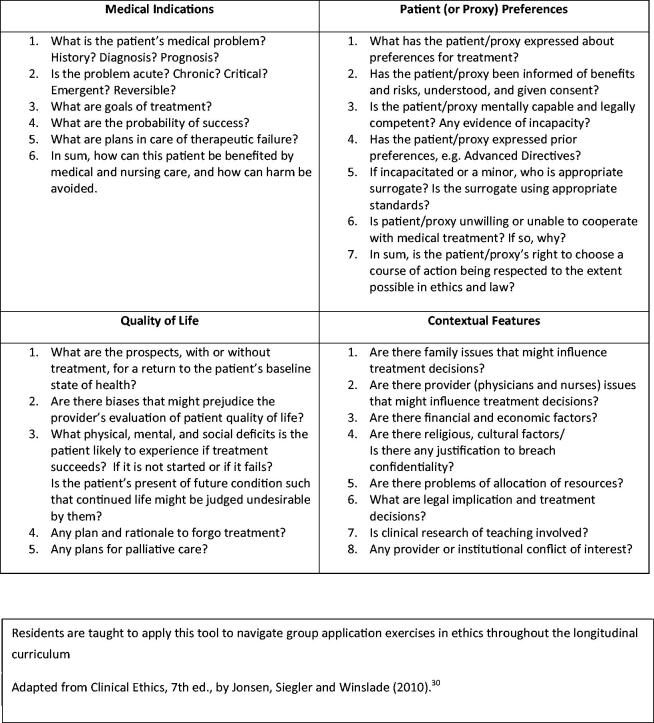
A TBL bioethics “Deliverable”: The four-box method of ethical analysis.

We integrated Jonsen et al.’s “Four-Box Method” (a widely utilised teaching tool in bioethics) into the curricular design (Figure 2) [30,31]. This method asks the learner to evaluate ethical cases *via* four essential “boxes,” analysed individually, and then balanced against one another to provide clarity in bioethical dilemmas. Integrating this method into TBL application exercises allowed residents to practice a consistent method of analysis, and to leave residency training with a “deliverable” skill, similar to other clinical reasoning methods and algorithms.

The core ABP ethics learning objectives provided the basis for the content of the TBL curriculum [1]. From these learning objectives, ten essential bioethics topics for residency education emerged, including basic principles of bioethics; professionalism topics such as social media use; and clinical ethics in the inpatient and outpatient settings (Table 1). Content is available at: https://bioethicstbl.org/.

**Table 1.** TBL bioethics sessions, content areas, and team application exercises.

TBL bioethics sessions	Examples of content assessed in iRAT/gRAT	Examples of multi-format TApp exercises
1. Introduction to Medical Ethics and the Four Box Method	Principles of bioethics such as autonomy, beneficence, nonmaleficence, justice; differences between adult and paediatric bioethics; Jonsen *et. al*’s “Four Box Method”	**Published ethical case** regarding a teenager’s wish to forgo chemotherapy for a treatable cancer against the advice of the medical team; Use of Four Box Method
2. Assent and Consent	Differences between assent and consent and clinical applications	**Ethical case** of new teenage mother with surrogate decision-making power in the neonatal intensive care unit
3. Professionalism and Social Media	Professionalism definitions; professional obligations to parents, families, and patients within social media; behaviour outside of work on social media	Several **simulated Facebook profiles** were created and learners were asked to critique them and discuss obligations of providers
4. Neonatal and Perinatal Ethics	End of life decision making in an infant with limited lifespan/Trisomy 13	**Use of video/YouTube case** of “Baby Thomas” and the Four Box Method: http://www.youtube.com/watch?v=ToNWquoXqJI. .
5. Spirituality in Medicine	Navigating conflicts between spiritual beliefs of providers and patients and medical outcomes	Discussion of real case at our institution of a devoutly Hindu family whose spiritual beliefs come into conflict with the advice of the medical team; **learners form their own “Ethics Committee”** and must come to a consensus decision using the Four Box Method
6. Paediatric Palliative Care	The ethical importance of palliative care; the nature of suffering; Palliative vs Hospice Care	**Role play:** Learners engage in a series of difficult conversations with an “SP” (“simulated parent”) of a seriously ill child; expert clinical faculty in paediatric palliative care participate in role play and discussion
7. Interpersonal Relationships	Professionalism; the role of hierarchy in the life of a resident; integrity	A clinical case of a resident learner who must respond to a clinical decision by an attending physician on the inpatient service they believe is wrong; **learners role play “scripts”** of how they would communicate and the ethical implications of those responses.
8. Paediatric Decision Making and the Best Interest Standard	Conflicts between parents and physicians/healthcare team; use and controversies surrounding the “best interest standard” in paediatrics	Learners analyse the famous recent bioethics case of “Charlie Gard.” They use the Four Box Method, are divided into teams, and then a **friendly mock debate** is conducted with brief speeches, cross examinations, and rebuttal periods.
9. Child Abuse, Intimate Partner Violence, and Toxic Stress	Impact of intimate partner violence on the developing child; ethical and social issues underlying family violence and the impact on health; legal and moral obligations of paediatricians to prevent family violence	Resident learners work through three ethical cases in large and small group settings: (1) a case of intimate partner violence with attention to **cultural stigma** and practices; (2) a publicly available **video case** of a female survivor of domestic violence and the effect on her children; (3) **learners listen to a graphic phone call** of a child calling 911 while violence is occurring in the home
10. Paediatric Research Ethics	The importance and dignity of the subject in research; the ethical obligations of the physician-researcher; The Belmont Report and Nuremberg Principles; the purpose of the IRB	Learners work in groups through **a simulated IRB application** and discuss problems and solutions, facilitated by expert faculty

Faculty facilitators were clinical content experts, trained in TBL and medical education by experts on our Task Force. Multiple-choice, face-valid questions were developed by faculty content experts for each TBL modules. Experienced ABP-format question writers, content experts, and Bioethics Education Task Force members reviewed questions to assure linkage to module content and learning outcomes.

### Phase 2: Curricular execution

TBL learning outcomes and preparatory resources were electronically distributed to residents at least one week prior to the in-person TBL activity. Examples of preparatory resources included textbook excerpts, scholarly articles, and AAP Policy Statements. Given the rigours of residency, a deliberate effort was made to limit readings to high-yield summaries, reviews, or policy statements, to keep preparation to an hour or less.

Upon arrival, residents were randomly assigned to small groups to promote diversity of training and perspectives. Then, each resident completed an iRAT, including 6-8 questions pertaining to the TBL pre-reading material and learning objectives.

Next, the assigned small groups of residents completed the gRAT by discussing the iRAT questions, with answers decided by consensus. Immediate feedback was provided when groups “scratched-off” immediate feedback assessment technique (IFAT) cards, revealing the correct answer to the group [32,33]. The facilitator(s) then led a large-group discussion focussing on challenging questions, and providing opportunities to discuss the rationale behind the answers.

Finally, the residents participated in a Team Application exercise (TApp), with clinical vignettes of an ethical/professionalism dilemma to analyse. Cases were presented in a variety of formats, including written narrative, live role-play, or with audiovisual resources [29]. We developed and adapted ethical cases from published national, local, or faculty experiences. Cases were anonymized, allowing residents to have “real world” examples to practice applying bioethics in a safe environment. Each small group used The Four-Box Method to analyse fundamental concepts underlying ethical decision-making, and then answered associated case vignette questions. Next, following traditional TBL methodology, teams simultaneously reported answers to the large group by holding up a card with an answer choice ("A," "B," etc.) printed on it, and then teams debated, defended, and appraised their answers in the large group setting [30]. This faculty moderated discussion asked provocative questions to promote critical thinking, and highlighted board-relevant and clinically pertinent principles of *practicing* bioethics.

### Phase 3: Assessment and evaluation

The iRATs were graded individually, with one point awarded for correct answers and zero points for incorrect answers. gRATs were graded for each group using the IFAT cards. All assessments were “low stakes,” since scores did not count towards formal competency assessment.

Statistical analysis included Kruskal-Wallis tests to examine for differences on iRAT and gRAT scores according to year of training and the paired t-test to compare mean iRAT and gRAT scores. gRAT score comparison between PGYs were further analysed using Chi square and a Dunn test. A *p*-value of <.05 was considered significant. The team statistician used SPSS for descriptive statistics and STATA for analysis [34,35].

At the end of each session the participants were asked to complete a post-session satisfaction survey. The post-session survey included a Likert scale (strongly disagree = 1 to strongly agree = 5), and respondents were asked if they completed the pre-TBL readings; these qualitative data have been published elsewhere, and we briefly summarise those results below.

## Results

Numbers of attendees for individual TBL sessions ranged from 24 to 44 for with a total of 348 resident encounters (Table 2).

**Table 2.** Individual results of Assurance Tests by trainee year.

	Total Attended (PGY 1-4)	PGY1		PGY2		PGY3		PGY4	
		iRAT	gRAT	iRAT	gRAT	iRAT	gRAT	iRAT	gRAT
N		122	134	115	120	75	74	13	9
TBL 1. Introduction to Medical Ethics and the Four Box Method	34	70% (13/13)	96% (13/13)	70% (11/11)	96% (11/11)	70% (9/9)	93% (9/9)	50% (1/1)	85% (1/1)
TBL 2. Assent and Consent	35	53% (15/16)	74% (15/16)	64% (9/9)	69% (8/9)	64% (10/10)	71% (10/10)	–	–
TBL 3. Professionalism and Social Media	26	68% (9/12)	92% (12/12)	67% (8/9)	90% (9/9)	56% (4/5)	86% (5/5)	–	–
TBL 4. NICU and Early Life	43	71% (15/21)	86% (21/21)	69% (11/13)	84% (13/13)	69% (6/8)	93% (8/8)	88% (1/1)	88% (1/1)
TBL 5. Spirituality in Medicine	24	89% (9/10)	94% (10/10)	75% (12/13)	89% (13/13)	63% (1/1)	94% (1/1)	–	–
TBL 6. Palliative Care	44	72% (44/44)	82% (44/44)	–	–	–	–	–	–
TBL 7. Interpersonal Relationships	30	82% (17/19)	95% (19/19)	86% (7/8)	95% (8/8)	81% (2/2)	100% (2/2)	75% (1/1)	88% (1/1)
TBL 8. Decision Making and the Best Interest Standard	40	–	–	–	–	79% (31/31)	95% (26/31)	74% (9/9)	100% (5/9)
TBL 9. Child Abuse, Inter-Partner Violence, and Toxic Stress	44	–	–	72% (44/44)	96% (44/44)			–	–
TBL 10. Research Ethics	28	–	–	79% (13/14)	88% (14/14)	78% (12/13)	89% (13/13)	63% (1/1)	81% (1/1)
Total		**72%**	**87%**	**72%**	**90%**	**73%**	**90%**	**72%**	**93%**

TBL: Team Based Learning; iRAT: Individual Readiness Assurance Test; gRAT: Group Readiness Assurance Test; PGY: Post-Graduate Year.

Our TBL curriculum had high satisfaction among the participants, with a mean overall satisfaction Likert scale rating across the 10 TBL modules of 4.42/5 [29]. Qualitative analysis of narrative comments by residents showed high levels of engagement, as well as appreciation of the content specifications, faculty facilitation of case-based group discussion, and the multi-modal approach to learning. They reported modest dissatisfaction with preparation time, multiple-choice questions, and pre-reading. Despite engagement during the TBLs, residents reported completing only 27.6% of the required readings (range: 14-47% across all 10 modules) [29].

The quantitative analysis showed a significant difference (*p* < .001) between iRAT mean scores (72%, SD 0.17) and gRAT mean scores (89%, SD 0.11). Average iRAT scores were not significantly different across PGYs (Chi square = 1.597, *p* = .907, df = 5). Average gRAT scores, however, were significantly different across PGY (Chi square = 18.392, *p* = .0025, df = 5) (Table 2). When evaluated further with a Dunn test, the mean gRAT scores increased with increasing post-graduate year. There was a statistical difference between intern (PGY-1) scores and senior residents. However, after intern year, scores did not differ statistically between cohorts of senior residents (Table 3).

**Table 3.** Comparison of average group readiness assurance test scores by trainee year.

	PGY1	PGY2	PGY3
PGY2	0.0001		
PGY3	0.0079	0.1979	
PGY4	0.0264	0.2891	0.1841

gRAT: Group Readiness Assurance Test; PGY: Post-Graduate Year.

## Discussion

This comprehensive, 3-year, TBL-based bioethics curriculum for paediatric residents is the first to be reported in the literature. It allotted multiple opportunities for residents to apply theoretical principles of bioethics to complex clinical scenarios they are likely to encounter in caring for children and families.

To our knowledge, this resident curriculum is one of the longest cohort studies in both TBL-specific and bioethics curricula to assess bioethical knowledge gains [7,27,28,36]. Prior studies evaluating TBL and bioethics addressed the needs of non-resident learners or focussed on a single, isolated TBL session [27,28]. This curriculum was also planned and grounded in L. Dee Fink’s theory of Significant Learning, a known successful approach to adult learning, also not found in the bioethics education literature [29,37,38]. Finally, despite the fact that inherent logistical and curricular pedagogic barriers within residency required us to deviate from the normal TBL process in the literature by incorporating neither peer review nor permanent teams, our results still demonstrated success both in knowledge gained and in overall satisfaction; we speculate that resident learners already brought significant team-building experience with them into bioethics TBL.

Significant increases in scores occurred between the iRAT and gRAT. This is consistent with TBL and other medical education literature which demonstrate that individual learning can be enhanced by group learning experiences [18,39–41]. This cohort study provides additional data supporting the effectiveness of TBL as a specific educational method and innovative approach to facilitate knowledge, provide an opportunity for application, and obtain immediate feedback through scoring practices and peer/faculty feedback. Of significance, while our curriculum was paediatric focussed, the planning, execution, and assessment methods could be utilised by any GME training program, by changing to specialty specific content.

Interestingly, resident iRAT scores did not vary with respect to their level of training. The lack of correlation between training level and iRAT scores highlights the possibility that some bioethical concepts may not be “naturally acquired” with merely additional years in clinical training. Acquisition and application of this knowledge seems best promoted by more formal educational opportunities, such as this curriculum [7]. The fact that gRAT scores improved with increasing seniority suggests that our residents developed their collaborative critical thinking skills as part of their education and medical experiences.

Given the constraints and heterogeneity of academic, clinical, and other professional demands, no single resident completed the entire curriculum. Encouraging use of the Four-Box Method provided residents with a useful framework to apply their knowledge of bioethics and then evaluate nuanced ethical dilemmas throughout their careers, regardless of how many sessions they attended [30]. Attending multiple sessions allowed residents to practice this method in a variety of clinical scenarios. Further studies are needed to evaluate this curriculum’s impact on the ability of participants to apply the Four-Box Method to new clinical bioethical dilemmas, and if there is a threshold effect on number of sessions required to gain this proficiency.

Implementing this curriculum highlighted the need for flexibility in resident education scheduling, where limited academic time forces GME training programs to choose between critical educational elements [11]. After the first year of the bioethics TBL curriculum, several structural changes occurred in overall resident education curriculum at the programmatic level, including changes in timing and program requirements, resulting in increased participation.

These unexpected changes highlighted several educational planning lessons: 1) anticipating change, curricula must be flexible enough to absorb and adapt while still achieving desired learning outcomes; 2) TBL is one methodology in which the length of the exercise can be altered significantly if teaching time is reduced (e.g. decreasing the number of iRAT/gRAT questions, shortening the Tapp) while still keeping group discussion robust; 3) in extreme circumstances, educators may need to cut important parts of the curriculum in order to save the larger goals of practical training in ethics; 4) our experience modifying and adapting the curriculum provided our team with the skills to quickly pivot during the COVID-19 pandemic. We continued to deliver our curriculum using remote/virtual platforms and “break out room” or small group functions. While we are collecting data on these adaptations, the successful use of “take home iRATs” in TBL was reported as early as 2018, and the Team-Based Learning Collaborative’s “White Paper” for best practices for online TBL was published prior to the pandemic [39,42]. Small post-pandemic studies have given us optimism that TBL used for bioethics can continue to improve knowledge, collaboration, and satisfaction now and in the future [43,44].

## Limitations

Sustained knowledge gains remain unknown and is a problem with bioethics education, which involves mastery of skills and attitudes to develop competence in practice. It is unknown whether the primary end-recipients—the patients and families—experience positive effects after receiving care from someone who has completed our training. However, TBL methods, including integration of “The Four-Box Method” reassure us that residents are equipped with both knowledge and a practical tool to navigate real-life bioethical issues.

iRAT and gRAT participant numbers did not consistently correlate within sessions due to unavoidable complexities within resident clinical schedules. Due to structural changes in the overall resident curriculum during the study period, not all TBLs were completed by residents from every PGY. “Make up” sessions were not feasible and recorded sessions for future passive viewing did not allow for group interaction.

We are fortunate that our institution has content experts in bioethics and a variety of subspecialties to facilitate TBL sessions. If outside institutions have fewer resources, one of the known advantages of TBL is the ability to train a small number of faculty in the facilitation of small and large group discussion [21].

Finally, we benefitted from significant support from paediatric leadership in prioritising bioethics education. Such support may not be available in every residency program but should be aspirational, given the mandate to include bioethics in GME and the importance of training physicians with fluency in applying the principles of bioethics to patient care.

## Future directions

Given the unique strengths of this curriculum (evidence-based, rooted in ABP content specifications, engaging, easily modified to virtual formats), we chose to make the entire curriculum available for free download at: https://bioethicstbl.org/. Through a grant from the Arnold P. Gold Foundation, our curriculum and website will be further developed to use TBL-based education to lay the groundwork for more humanistic patient care [45]. This includes engaging other paediatric residency programs to utilise this curricular work, developing “train the trainer” opportunities for faculty, and creating interprofessional bioethics TBLs to include social workers, nurses, and psychologists. The effectiveness demonstrated here provides a future opportunity to fill another gap in the literature—to compare learning outcomes, satisfaction, and behaviour between this curricula and other extant longitudinal curricula (e.g. the AAP online curriculum, carefully implemented) to determine best practices in paediatric bioethics education. Finally, as this study was completed prior to the COVID-19 pandemic, we are evaluating the effectiveness of our transition to a virtual education format. Anecdotally, it has been well-received by both faculty and participants.

## Conclusions

This comprehensive, innovative, TBL-based bioethics curriculum is a first in bioethics education and in GME residency training. Our curriculum led to improvements in knowledge; provided trainees with an opportunity to apply practical collaborative skills in controlled peer-group settings; led to high learner satisfaction; and contained flexible components that can be adapted to different institutions, provider types, learning environments, programmatic structural changes, and even pandemics. These data indicate that the utilisation of TBL in bioethics education with the integration of practical tools such as The Four-Box Method, may influence future ethical behaviour in medical residents by providing engaging, evidence- and practice-based learning.

## Data Availability

The data that support the findings of this study are available from the corresponding author, Dr. Fernandes, upon reasonable request.
